# Flowering Dynamics and Pollinator Visitation of Oilseed Echium (*Echium plantagineum*)

**DOI:** 10.1371/journal.pone.0113556

**Published:** 2014-11-26

**Authors:** Carrie A. Eberle, Frank Forcella, Russ Gesch, Sharon Weyers, Dean Peterson, James Eklund

**Affiliations:** United States Department of Agriculture-Agricultural Research Service, North Central Soil Conservation Research Lab, Morris, Minnesota, United States of America; Northwest A&F University, China

## Abstract

Echium (*Echium plantagineum* L.) is an alternative oilseed crop in summer-wet temperate regions that provides floral resources to pollinators. Its seed oil is rich in omega-3 fatty acids, such as stearidonic acid, which is desired highly by the cosmetic industry. Seeds were sown in field plots over three years in western Minnesota in spring (early-sown) or early summer (late-sown), and flower abundance, pollinator visitation, and seed yields were studied. Initial flowering commenced 41 to 55 d after sowing, and anthesis duration (first flowering to harvest) was 34 to 70 d. Late sowing dates delayed anthesis, but increased the intensity of visitation by pollinators. Cumulative flower densities ranged from 1 to 4.5 billion ha^−1^. Flowers attracted numerous honey bees (*Apis mellifera* L.), as many as 35 per minute of observation, which represented about 50% of all insect visitors. Early-sown echium produced seed yields up to 750 kg ha^−1^, which were 2–29 times higher than those of late-sown echium. Early sowing of echium in Minnesota provides abundant floral resources for pollinators for up to two months and simultaneously produces seed yields whose profits rival those of corn (*Zea mays* L.).

## Introduction

Echium (*Echium plantagineum* L., Boraginaceae) is a winter annual weed in Mediterranean climates [1]. However, high concentrations of desirable fatty acids occur in its seeds, and its flowers are attractive to pollinators. Given its history as a weed elsewhere, attempts to grow echium as a crop are uncommon. The combined benefits of commercially valuable fatty acids coupled with the growing interest in supporting pollinators warrants further exploration of agricultural production of echium.

Echium seeds contain about 27% oil that is enriched with high levels of stearidonic acid (SDA) and gamma-linolenic acid (GLA) [2]. These fatty acids are rare in plants and highly valued in the health and personal care industries. Stearidonic acid, in particular, is an essential ingredient in anti-wrinkle cosmetics, and both SDA and GLA provide health benefits analogous to fish oils [3].

In addition to its seed oil value, echium is highly attractive to pollinating insects, especially honey bees. Echium is a common melliferous (honey producing) plant native to the Mediterranean Basin and naturalized throughout southern Australia, where it is used widely for honey production [4],[5],[6]. Nearly 15% of Australian honey is derived from echium [7]. Regions that support high densities of honey bee colonies and/or desire to augment nutritional resources for native pollinators have critical needs for such plants. Despite echium's ability to support pollinators, its history as a weed has limited its use as a crop.

Reports on growing echium as a crop are rare. Berti et al. [8] reported seed yields of 115 to 617 kg ha^−1^ for autumn-sown echium in Chillan Province, Chile. Echium also has been grown successfully as a spring-sown crop in the United Kingdom, with yields of 200 to 300 kg ha^−1^ [9]; and at four locations in North Dakota, where yields ranged from 63 to 425 kg ha^−1^ and averaged 251±29.5 kg ha^−1^ across 12 site-years [10]. No weedy tendencies of echium in or near the North Dakota experimental sites were observed. Relationships between seed yields and growing season temperatures or rainfall totals (North Dakota Agricultural Weather Network values) in North Dakota were not obvious in our examination of the data from Berti et al. [10], which indicated that the short growing season and relatively low rainfall of the Northern Great Plains did not restrict echium from achieving relatively high seed yields in some sites and years.

Our interest in echium arose from a desire to extend floral resources for pollinators *via* high-value oilseed crops in the Upper Midwest and Northern Great Plains of the USA. This region supplies a large proportion of the nation's transient honey bee colonies, and such colonies suffer high yearly losses. Summer-time nutrition is thought to play an important role for subsequent over-winter survival and vigor during the colonies' fruit and nut pollination activities in California and other southern locations [11],[12]. Echium production in the Upper Midwest and Northern Great Plains may help solve this dilemma. Consequently, we examined the effects of echium sowing date on flowering dynamics, pollinator visitations, and seed yields, and the potential energy (nectar) an echium crop may provide to pollinators. Based on three years of data we were able to document extraordinary levels of flower production and pollinator visitation, as well as provide guidance regarding planting dates that assures both high seed yields and pollinator resources.

## Materials and Methods

### 2.1 Plot establishment

Experiments were performed at the USDA-ARS Swan Lake Research Farm, Stevens County, MN (45.68°N, 95.80°W) on a Barnes loam soil (fine-silty, mixed, super-active, frigid Calcic Hapludoll) during 2011, 2012, and 2013. Treatments (planting dates) were arranged in a randomized complete block design with three replications in 2011 and 2012. Plots were 3.1 by 12.2 m. In 2013, data were collected from four replications of each of two separate but proximal experiments involving nine oilseed crops, including echium. In the first experiment (May-sowing) the plots were 6.1 by 18.3 m, and in the other (June and July sowings) the plots were 2.4 by 3.1 m. The variability in plot sizes is unlikely to have influenced flowering dynamics, but potentially could have affected pollinator visitation and seed yields.

Previous crops were soybean (*Glycine max* L. [Merr]) in 2010 and wheat (*Triticum aestivum* L.) otherwise. Fertilizer was applied at a rate of 77-33-33 kg ha^−1^ of N-P-K and incorporated with the field cultivator or a no-till drill at sowing. Fertilizer applications were based on those recommended in the NNFCC crop fact sheet [9]. For weed control, trifluralin herbicide (2,6-dinitro-*N,N-*dipropyl-4-(trifluoromethyl)aniline) was applied preplanting at 0.75 kg ai ha^−1^ through a 3.1-m tractor-mounted boom that delivered 187 L ha^−1^ at 207 kPa pressure. Weeds that escaped herbicide control were removed by hand.

Echium seeds were obtained originally from Technology Crops International (TCI) in 2008. Harvested seeds were cleaned and resown each year thereafter. Seeds were sown 1.5 to 2.5 cm deep in rows spaced by 20 cm and at a rate of 11 to 17 kg seed ha^−1^ depending upon viability (TCI growing guide for echium recommends a seeding rate of 11 kg viable seed ha^−1^ based on germination percent of seed stocks).

Sowing dates were May 26 and July 7, 2011; April 25 and June 15, 2012; and May 14, June 13, and July 8, 2013 (table 1). Early sowing of echium was completed as soon as field conditions allowed each spring. Late sowing was as near to 7 weeks after early sowing as was possible based on weather conditions. Late sowing of echium was done 6, 7, and 8 weeks after early sowing in 2011, 2012, and 2013 respectively. An additional mid-season sowing date was planted on June 13, 2013 (4 weeks after the early sowing) in order to evaluate if seed yields could be improved relative to the late sowing and to provide uninterrupted floral resources to pollinators from early summer to autumn.

**Table 1:** Echium sowing date and associated flowering dynamics, cumulative rainfall (from sowing to harvest), cumulative thermal time of air (from sowing to harvest), and seed yield.

Year	Planting Time	Time to first flower (days)	Anthesis Duration (days)	Rainfall (mm)	Thermal time (Cum °d)	Seed Yield (kg ha^−1^±SD)
2011	Early	42	70	304	2352	773±181
	Late	41	69	245	2015	27±6
2012	Early	54	55	247	2179	327±20
	Late	55	34	148	1992	85±19
2013	Early	44	57	292	2003	430±151
	Mid	36	54	238	1978	387±33
	Late	46	48	145	1867	258±125

Weather data were collected throughout the growing season at the Swan Lake Research Farm Weather Station (http://www.ars.usda.gov/Services/docs.htm?docid=3512). Cumulative soil thermal times (at 5 cm depth; base temperature, 0°) between January 1 and the sowing dates each year were 474 and 1270 (2011), 393 and 1268 (2012), and 201, 659, 1238 (2013).

### 2.2 Flowering Dynamics

Percent ground cover of echium flowers (flower area) was estimated visually in each plot from the time open flowers were first visible through the end of anthesis for all three years. Visual estimates were made by two observers by viewing the crop from above and approximating the percent of the total area that was covered by flowers. Cumulative flower area was estimated by calculating the areas under the curves throughout anthesis. These integrals represent the coverage time (%t) of flower area. Anthesis duration was measured from the start of flowering to the time of swathing; because echium is an indeterminate crop, anthesis persisted until the crop was harvested.

In 2013, number of open flowers were measured once per week during anthesis using the Batcheler corrected point distance estimation [13]. A 1.8 m transect rope with six pre-marked points 30 cm apart was used to establish three transects within each plot. The distance from each point to the center of the nearest open flower was measured, as was the distance from the center of the first open flower to the center of the next nearest open flower. Measurements were taken on at least five different dates throughout anthesis for each planting date. During field measurements, the maximum search distance for a flower was set to 15 cm to ensure that individual flowers were not included more than once. During data analysis, the maximum search distance for each date was adjusted to exclude approximately 30% of the flowers, as per Remple [13]. Calculations of flower density (f ha^−1^) were performed using Transect Point Density software [14]. The average flower density within each planting date was plotted by the day of year. The sum of flowers produced per hectare throughout anthesis (∑f ha^−1^) was calculated by integrating the area under the curve.

### 2.3 Nectar Collection and Analysis

Nectar analysis was performed on early-sown echium flowers on July 16 and August 7, 2013, following protocols described by Corbet et al. [15]. On the former date the mean temperature was 26.4°C, mean solar radiation was 0.33 kwh/m^2^ (PAR = 479), with a mean RH of 71%, and no measurable rainfall 24 h before nectar was removed. On the latter date the mean temperature was 18.2°C, mean solar radiation was 0.30 kwh/m^2^ (PAR = 525), with a mean RH of 74%, and 0.64 cm of rainfall 24 h before nectar was removed. Nectar was extracted from 10 open flowers using a 1, 5, or 10 µL microcapillary. The length of the nectar column in the microcapillary was measured immediately after extraction. Degree Brix (°Bx; % sucrose) was recorded for each flower using an Eclipse low volume handheld refractometer (Bellingham + Stanley Inc, GA, USA). “Standing crops” of nectar were removed at three time points: 900, 1200, and 1500 h, from 10 different flowers at each time, and the flowers were tagged and isolated with insect exclusion bags. After 2 h of exclusion, the flowers were resampled for nectar volume and °Bx. Nectar volume was calculated by dividing the length of the nectar column by the total length of the microcapillary and multiplying by the microcapillary volume. Sugar content was calculated from the Corbet et al. [15] equation for nectar sucrose content:


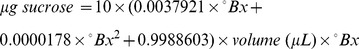


giving the total sucrose produced in 2 h by a single echium flower. The µg of sucrose produced was averaged across the two sampling dates for the 10 flowers sampled during each time interval. The daily sucrose production per echium flower was approximated by summing the average µg of sucrose produced during each of the three time intervals. The sum of flowers produced per hectare throughout anthesis was multiplied by the daily sucrose production of a single echium flower to calculate the potential sucrose production of a hectare of echium throughout anthesis.

### 2.4 Insect Counts

Pollinator visitations to the plots were recorded from the time open flowers were first visible through the end of anthesis. Visitation was documented by investigators walking along the length of each plot for 1–2 minutes and listing the number of individuals observed in each of the following insect categories: honey bee, bumble bee, other bee, fly, butterfly, and other insects. Insect counts were conducted between 1100 and 1400 h on rainless days, with wind speeds <7 m s^−1^, when the temperature >5°C and the sky was ≥50% clear or when the temperature was >10°C with any sky cover. All insect counts were divided by the number of observers and the minutes of each observation period giving number of insects observed by one observer in 1 min. Integrating under the curves of pollinator-time relationships allowed calculation of pollinator visitation time (pvt) throughout anthesis. A ratio of insect visitation to flower area was derived by dividing pvt by %t for each planting date. A larger value indicates more insects per flower area. The 52-ha research farm harbored one commercial-grade honey bee colony in 2011 and 2012, and four colonies in 2013. In addition, a commercial apiary with 34 hives was located within two miles of the research farm, which supported consistent bee visitation each year. Otherwise, the research farm was surrounded by narrow strips of semi-natural vegetation (tree-lined lakeshore, woodland, wetlands, and grassed fence lines and roadsides), which likely provided nesting habitat for native pollinators.

### 2.5 Seed Harvest

Echium seeds were harvested by swathing sections in each plot on September 15 and October 25, 2011; August 12 and September 12, 2012; for the early and late planted plots, respectively and August 23, 2013 for the early planted plot. The 2013 mid planted plot was harvested on September 11 by straight combining without swathing. The 2013 late planted plot was harvested on October 10 by hand harvesting two rows, allowing the plant material to dry, and hand threshing the seed. Variation in harvesting techniques in 2013 was unavoidable due to unforeseen problems with machinery and labor availability. Swaths typically were 1.5 m wide and the length of the plot. Swathing and direct harvesting occurred when the bottom third of former flowers along branches of the cyme-type infructescence had black seeds (dried and fully mature), the middle third had grayish seeds (physiologically mature), and the upper third had green seeds or still-maturing flowers [16]. Echium does not have seed pods; instead, its fruits are small nutlets, typically four per flower, whose dark colors are easily visible at the base of the mature tubular calyx, the corolla having abscised earlier. Swaths were combined or hand threshed (small plots in 2013). Seeds were dried and chaff removed prior to calculating yield based upon 10% seed moisture [16].

### 2.6 Statistical Analysis

Estimation of open flower density was done using Transect Point Density software [14], which calculates a corrected point density (CPD, open flower density) based on point to object densities and adjusts for clustering within the plots giving a 95% confidence limit. There were a total of 72 points used to calculate the CPD for each date. Standard error was calculated by subtracting the lower confidence limit from the upper confidence limit and dividing by the t value multiplied by two. Multiple regression modeling was performed using stepwise selection in SAS 9.3 statistical software. Predictor variables for anthesis period were modeled using sowing date (early, mid, late), days to flower (days from sowing to first flower), anthesis-rainfall, vegetative-rainfall, anthesis thermal time (from first flower to harvest), and total thermal time. Yield was modeled using sowing date, growing season days (from sowing to swathing), thermal time, anthesis-rainfall, and vegetative-rainfall. Only variables with a *P*-value <0.15 were included in the model. Standardized regression coefficients (SRC) are computed in SAS 9.3 by dividing a parameter estimate by the ratio of the sample standard deviation of the dependent variable to the sample standard deviation of the regressor (SAS 9.3). The SRC indicates how many standard deviations a given variable will change per standard deviation increase in the predictor variable independent of scale.

## Results and Discussion

### 3.1 Flowering dynamics

Time from sowing to first flower averaged 45 d and varied little across sowing dates. Flowering ended at swathing, typically in late August but extended into early October for late sowings. The average of 55 d for anthesis duration in the relatively wet climate of Minnesota was similar to flowering of cultivated and weedy populations in Mediterranean-like environments. The flowering period for echium is about 30 to 40 d in its native Mediterranean Basin [17], whereas in weedy populations in southern Australia, the duration of flowering is approximately 60 days [18]. The anthesis duration of potted plants under natural light conditions was 71 d in southern Australia [19].

**Figure 1:**
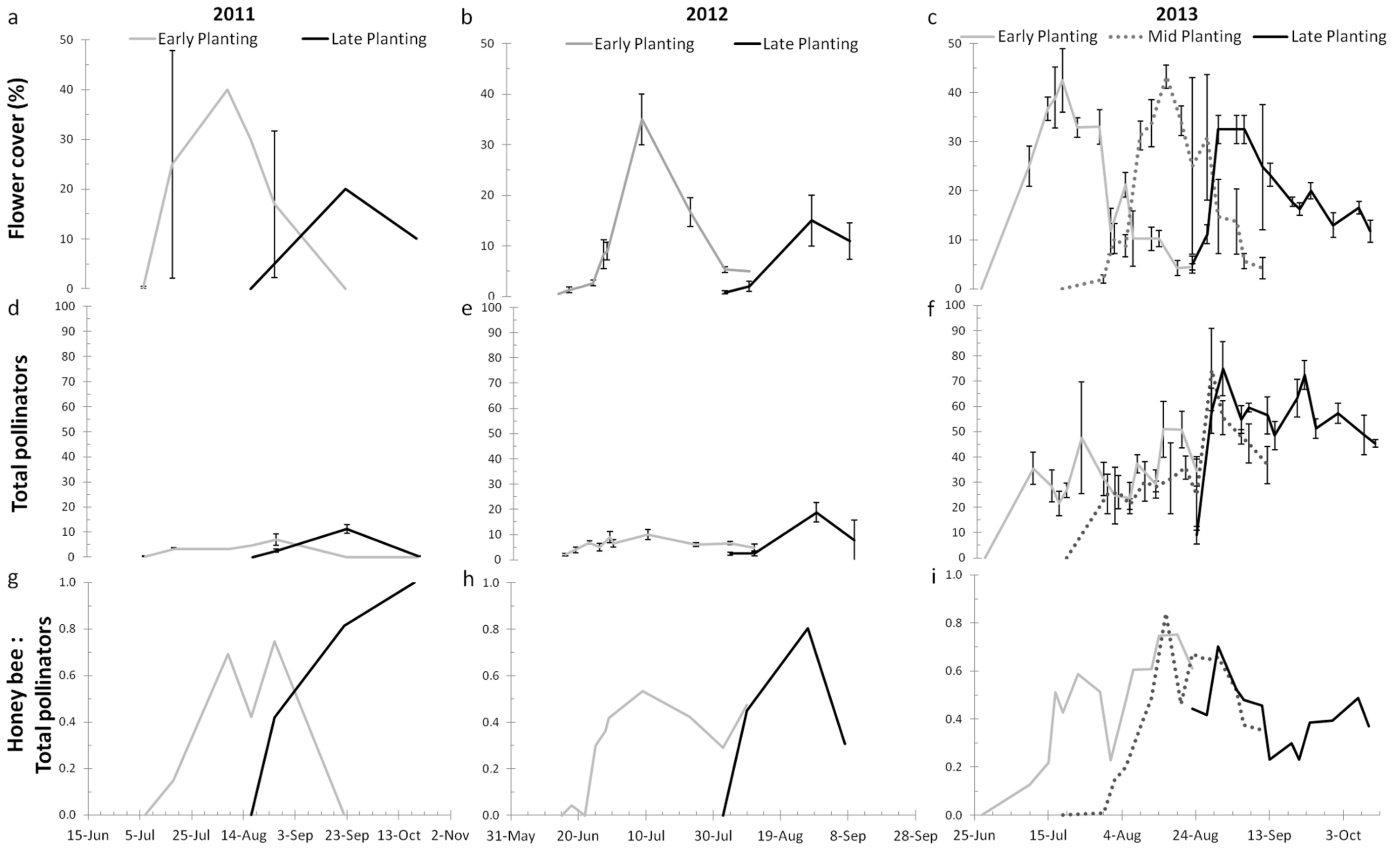
Dynamics of open flower cover and total pollinator abundances. Percent flower cover of early- mid- and late-sown echium in 2011 (a), 2012 (b), and 2013 (c) and total pollinators observed per min per observer (d, e, and f). Proportions of honey bees compared to total pollinators in 2011 (g), 2012 (h), and 2013 (i). Error bars (a–f) are standard deviation.

Flowering peaked at or near 40% cover within 2 to 3 weeks of the start of flowering, and remained above 10% for most of anthesis (figure 1a–c). In 2011, the anthesis duration, was about 70 d for both planting dates (table 1). In contrast, flowering duration was shorter during 2012 and 2013 ranging from 34–57 d. Stepwise selection multiple regression modeling indicated days to first flower, anthesis-rainfall, and vegetative-rainfall were significant variables in determining anthesis duration, with negative, positive, and positive coefficients, respectively (table 2). Thus, the lower the number of days to first flower from sowing, the longer the duration of flowering. Rainfall during anthesis was most strongly associated with increased anthesis time (SRC  = 0.42; table 2): as anthesis rainfall increased so did the number of anthesis days. Therefore, drought conditions during July 2012, during anthesis of the late-planted echium, likely contributed to the short duration of flowering that year. Higher rainfall during vegetative growth also increased flowering duration.

**Table 2:** Stepwise multiple regression model of anthesis duration using days to flower (DTF), anthesis rainfall (ARF), and vegetative rainfall (VRF). R^2^ = 0.77.

Variable	DF	Parameter Estimate	Standard Error	*t* Value	Pr> *|t|*	SRC^a^
Intercept	1	60.89	12.7	4.79	0.0001	0
DTF	1	−0.64	0.21	−3.11	0.0055	−0.38
ARF	1	0.21	0.07	3.18	0.0048	0.42
VRF	1	0.03	0.01	2.56	0.0187	0.37

a SRC, Stepwise regression coefficient.

Coverage times of flower area (%t) for early- and late-sowing dates were 1577 and 765%t in 2011, 769 and 535%t in 2012, and 1113 and 972%t in 2013 (table 3). Thus, late sowings had 49%, 70% and 87% of the flower coverage time as early sowings of echium in 2011, 2012, and 2013, respectively. The mid-sowing date for echium in 2013 had 929%t, which was 83% of the early-sown echium flower coverage time. For comparison with standard commodity crops, the 2013 flower coverage time for corn was 9%t and that for soybean was 2%t. These values are 57 and 215 times smaller than even the lowest %t of echium. Therefore, the combined extent and duration of flower availability to pollinators was much less in corn and soybean than in echium.

**Table 3:** Flower coverage time (%t), pollinator visitation time (pvt), pollinator visitiation intensity (pvt %t^−1^), sum of flowers produced per hectare (∑f ha^−1^), sucrose per hectare per year (s h^−1^ y^−1^), and ratio of pollinator visitation time to flower coverage time (pvt %t^−1^).

Year	Planting time	%t	pvt	pvt %t^−1^	∑f ha^−1^ (billion)	s h^−1^ y^−1^ (kg)
2011	Early	1577	271	0.17		
	Late	765	356	0.47		
2012	Early	769	376	0.50		
	Late	535	897	1.68		
2013	Early	1113	1787	1.61	1.01	641
	Mid	929	1774	1.91	4.59	2911
	Late	972	2662	2.74	0.98	609

### 3.2 Pollinator Food Provisions

**Figure 2:**
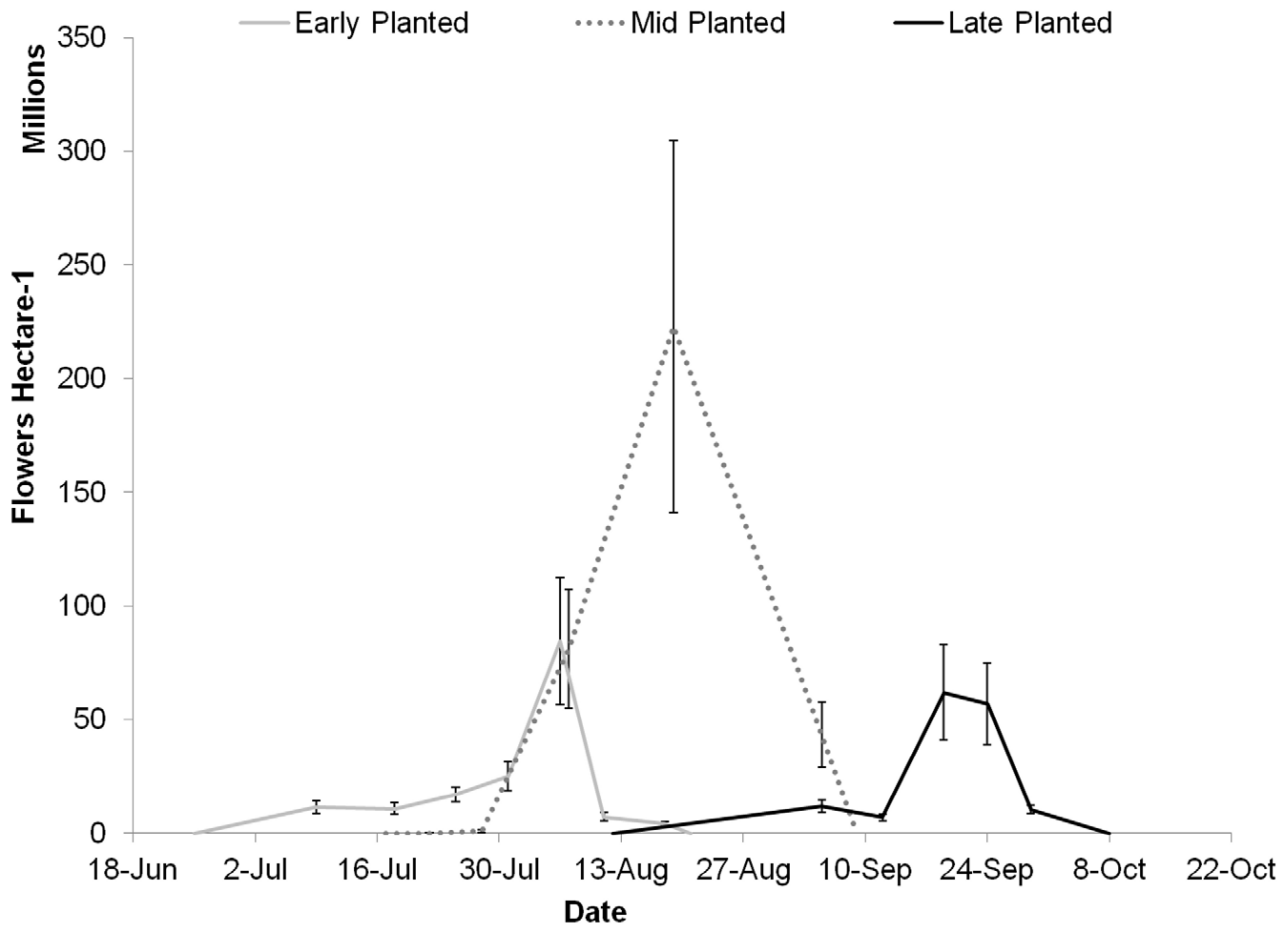
Flower density of echium throughout anthesis. Open flowers ha^−1^ in early, mid, and late sown echium during 2013. Error bars are standard error.

The sum density of flowers produced per hectare over the anthesis period (figure 2) was 1.01, 4.59, and 0.96 billion ∑f ha^−1^ for the early, mid, and late sown echium in 2013 (table 3). Although, the early sowing date had the highest flower coverage time (%t) of the three planting dates, the mid planting date had the highest sum flower density throughout the anthesis period. For comparison, a very high and prolific soybean population of 500,000 plants ha^−1^ bearing 800 flowers plant^−1^ [20] would produce 400 million flowers ha^−1^, which is two to ten times lower than that of echium.

**Figure 3:**
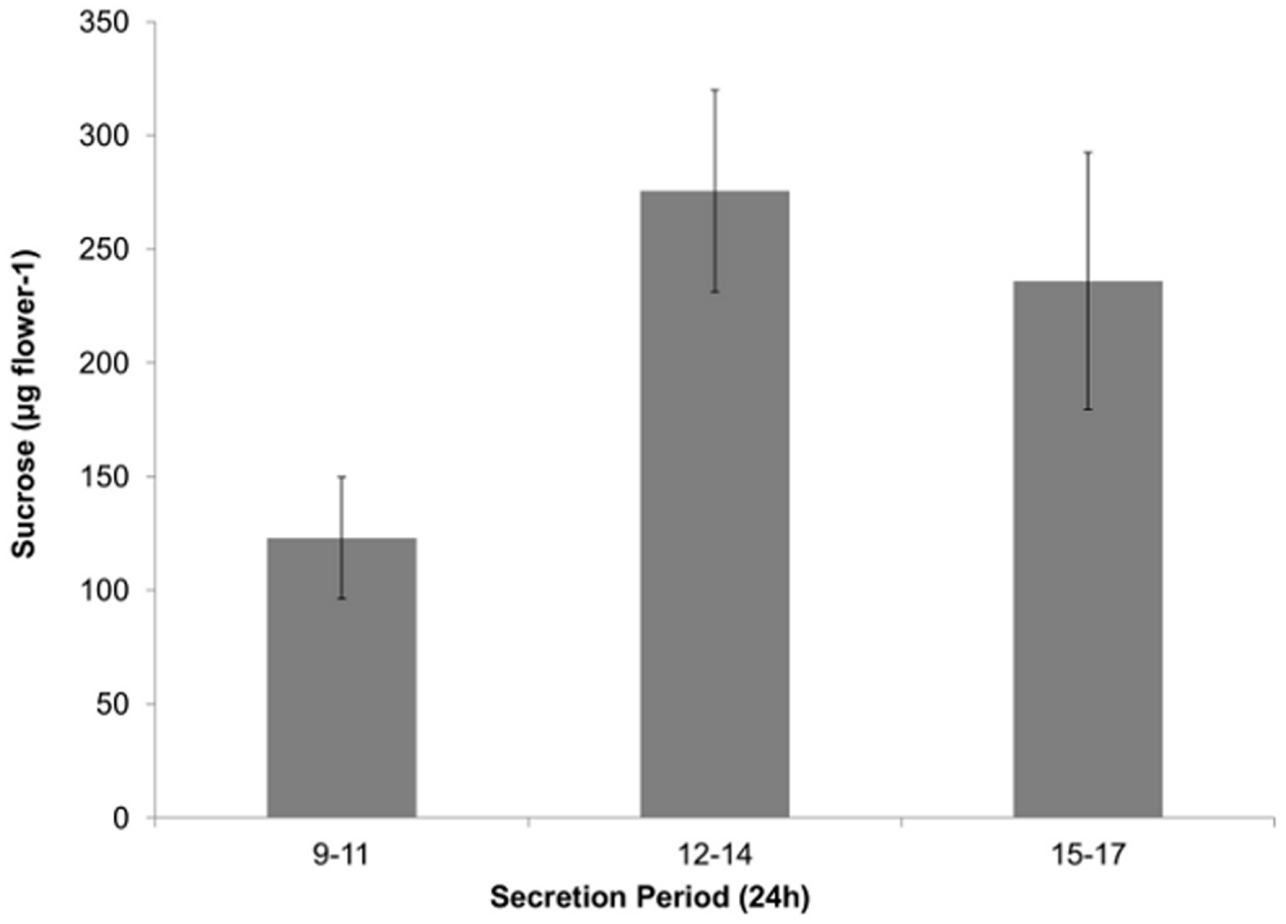
Sucrose produced by echium flowers throughout a day. Average sucrose (μg) in nectar secreted from 900-1100 h, 1200-1400 h, and 1500-1700 h during two days in the 2013 growing season. Sucrose quantity was based on volume and ○Brix of nectar extracted from individual flowers 2 hours after standing nectar was removed from the flower. Average ± S.E. sucrose amount is for 20 flowers at each time point.

In 2013, nectar was extracted from flowers at three time points on two dates during peak flowering, and nectar sucrose production was calculated on a 2-h basis for each time point (figure 3). Echium flowers produced nectar from 900 to 1700 h, providing a food resource to foraging insects from dawn to dusk. Sucrose production for each time point was not significantly different between the two sampling dates, despite varying weather conditions [21]. The cumulative nectar sucrose produced by an individual echium flower throughout the day was equivalent to 635 µg of sucrose d^−1^, with peak production from 12:00–14:00 h. The sum densities of flowers throughout anthesis was used to estimate the potential energy each sowing-date of echium would provide to pollinators by multiplying the cumulative nectar sucrose produced by a single flower in a day by the sum of flowers produced throughout anthesis. This estimate gave a potential of 641, 2911, and 609 kg of sucrose ha^−1^ season^−1^ produced by the early, mid, and late planted echium, respectively (table 3).

A healthy honey bee colony requires approximately 100–200 kg yr^−1^ of sugar [22],[23],[24]. The TCI Growing Guide [16] for echium suggests placement of two honey bee hives per hectare to ensure good seed set. Our results indicated a hectare of early or late planted echium in 2013 had the potential to support 3–6 hives for an entire year while the mid-sown echium could have supported 15–29 hives on its nectar production. This level of colony support assumes (unrealistically) that the flowers produce nectar equally throughout anthesis and that honey bees forage the full nectar flow on every day throughout anthesis.

Corbet and Delfosse [4] reported a nectar sugar yield of 300 kg ha^−1^ yr^−1^, which is about half of what we predicted for our early sown crop in 2013. They also reported that nectar secretion in echium is affected by density of flowers, with higher densities of flowers having lower secretion rates. We extracted nectar before peak flower density was reached in the 2013 early sown crop, thus nectar production later in the season may have decreased as the flower density increased. These assumptions likely led to overestimations of sucrose production. More detailed investigations into nectar production throughout anthesis, as well as pollen production, and foraging by honey bees, are needed to give a more accurate recommendation for hive densities that can be supported near echium fields. Regardless of inaccuracies, however, the results indicate that echium not only can support high densities of honey bee colonies, but can supply most of their annual energy needs during a single one- to two-month flowering/foraging season.

Comparison of sugar production values by echium to those of soybean reveals the potential value of having echium on the landscape. Erickson [25],[26] examined nectar production of several soybean varieties, but emphasized ‘Hark’ (MG I), which was attractive to honey bees. ‘Hark’ produced 0.01±0.002 µL flower^−1^ of nectar, and the sugar concentration of soybean nectar extracted directly from bee's stomachs was 36.2±1.94%, giving approximately 5 µg sugar per flower. Thus, at a flower density of 100–800 flowers per plant [20],[27] and plant density of 0.5 million ha^−1^ we calculated approximately 0.5 to 4 L ha^−1^ of nectar and 0.25 to 2 kg ha^−1^ of sugar would be available to nectar-gathering insects. Similarly, Severson and Erickson [28] reported soybean nectar carbohydrate (sugar) levels of 16 to 134 µg flower^−1^ for a range of cultivars. These values convert to 2 to 54 kg ha^−1^ of sugar at the same flower density mentioned above. In brief, even the highest value of nectar sugar production for soybean is an order of magnitude lower than that for echium.

### 3.3 Pollinator Visitations

**Figure 4:**
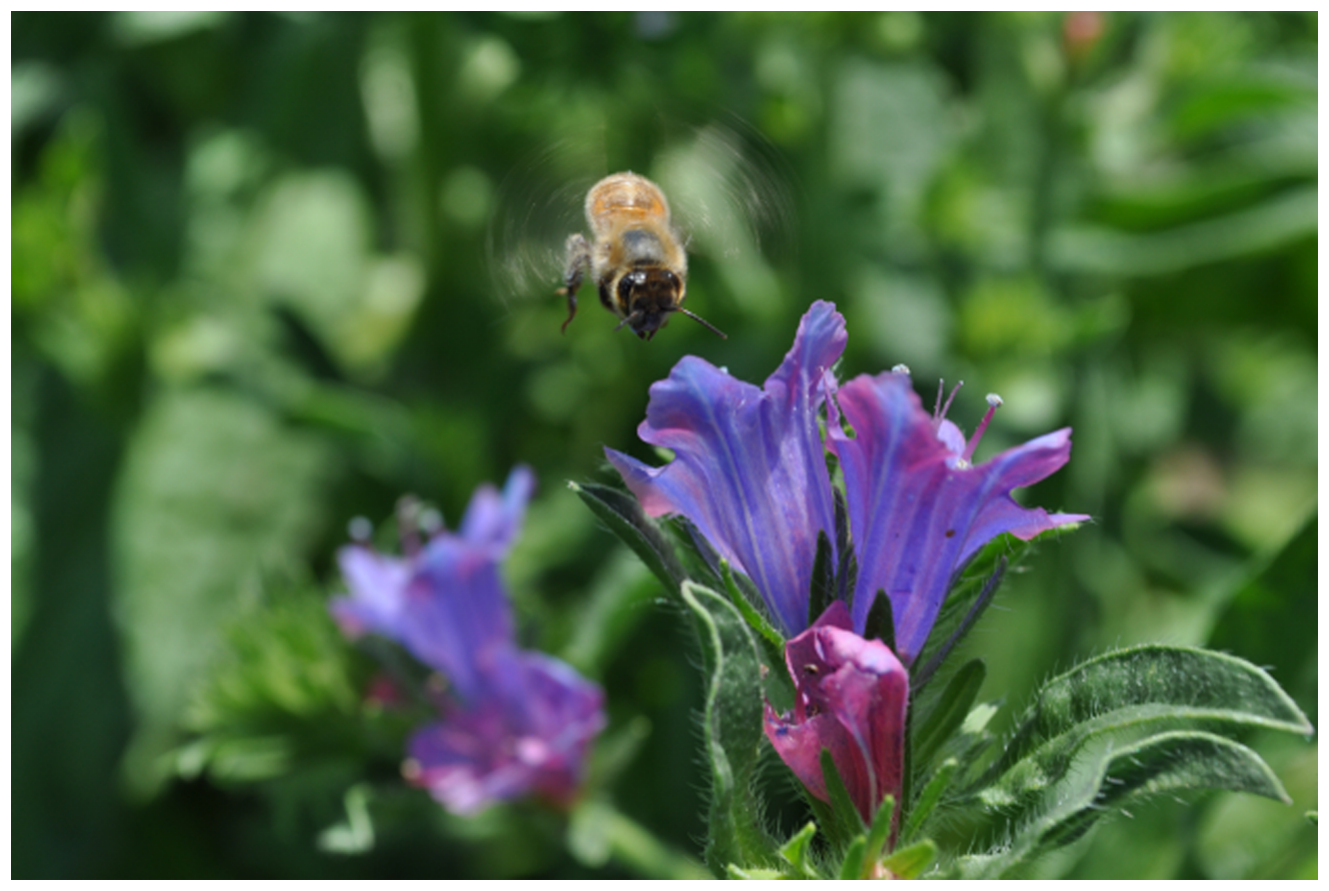
Honey bee visiting an echium flower. Photo by James Eklund.

Insect visitors were present immediately upon initial observations of open flowers (figure 1 d–f and figure 4). At times of peak flowering, 10 to 70 pollinators min^−1^ were observed. The numbers of pollinators visiting echium were much higher than those reported for other oilseed crops, such as *Thlaspi arvense* L. (1.1 pollinators min^−1^) and *Camelina sativa* L. Crantz. (3.1 pollinators min^1^) [29], but span the values for *Brassica napus* L. (20 pollinators min^−1^) [30] and *B. rapa* L. (10 to 50 pollinators min^−1^) [31].

Pollinator visitation times (pvt) were 271 and 356 pvt in 2011, 376 and 897 pvt in 2012, and 1787, 1774, and 2662 pvt in 2013 (table 3) for early and late planting dates, respectively. In 2011, 2012, and 2013 the late planted echium had 132%, 239%, and 149% higher pvt than the early planting dates. These data are surprising considering the %t was reduced in the later plantings. The ratio of pollinator visitation time to flower coverage time (pvt %t^−1^) increased in the later sown echium relative to the early sown echium each year (table 3). Results suggest that either insect populations early in the flowering season were too low to make full use of available echium flowers or, less likely, flowers of other plant species in the local area early in the season may have distracted pollinators from echium.

Honey bees comprised the majority of pollinators often representing more than half of the observed insects (figure 1 g–i). Exceptions were at the beginning of the anthesis periods for each year and sowing date treatment when the flower area was very low. Presumably, at those times honey bee scouts had not yet found the echium flowers. Other insects, especially hoverflies (Syrphidae), were primary floral visitors during the earliest and latest observations of the flowering seasons. The additional three honey bee hives in 2013 may have contributed partially to the increased pvt; however, the other insect groups also had increased numbers in 2013 relative to 2011 and 2012, indicating that the increased pvt was not due solely to the increased presence of honey bees.

As a comparison to commodity crops, the pvt for corn and soybean in 2013 were 177 and 378, which were four and two times less than the lowest pvt for echium across all years, which shows that presence of echium provides valuable insect forage in a landscape dominated by corn and soybean. Honey bee visits to corn and soybean only accounted for 5 and 4%, respectively, of all insects observed, which was over 8 times lower than the average proportion of honey bee visits of 42% in echium (data not shown).

A caveat regarding echium as a forage resource for honey bees is that its pollen contains pyrrolizidine alkaloids, which is potentially harmful to mammals [32],[33]. Alkaloids from the pollen may infuse into nectar in beehives and, thereby, contaminate honey. Fortunately, human exposure to contaminated honey is unlikely [34],[35], and the plants are common sources of safe honey in southern Australia [7] where echium is abundant. Consequently, use of echium as a melliferous plant on landscapes of the Upper Midwest and Northern Great Plains should not be problematic.

### 3.4 Seed Yields

The average seed yield across all planting dates and years was 330±49 kg ha^−1^. Early-sown echium had 285, 386, and 167% higher yield than late-sown echium in 2011, 2012, and 2013, respectively (table 1). Across the three years, early planting dates averaged 510 kg ha^−1^, and late planting dates averaged 123 kg ha^−1^. The absolute highest yield was observed for the early-sowing date in 2011 and the lowest yield occurred during the drought year of 2012 for the late-sowing date.

Seed yield modeled with stepwise selection methods indicated growing season days, vegetative rainfall, and anthesis rainfall were all significant terms in predicting yield (R^2^ = 0.85, table 4). Thermal time was not a significant factor and not included in the final model. Rainfall during anthesis was positively associated with yield and had the highest standardized estimate and, therefore, the most influence on yield. Growing season days were correlated negatively with yield, meaning a longer growing period resulted in lower yield. This correlation may be explained partially by increased seed loss due to shattering as the echium crop matured longer.

**Table 4:** Stepwise multiple regression model of seed yield using growing season days (GD), anthesis rainfall (ARF) and vegetative rainfall (VRF). R^2^ = 0.85.

Variable	DF	Parameter Estimate	Standard Error	t Value	Pr> |t|	SRC
Intercept	1	1546.93	308.64	5.01	<0.001	0
GD	1	−19.89	3.66	−5.43	<0.001	−0.76
ARF	1	12.26	1.43	8.55	<0.001	1.11
VRF	1	0.66	0.2	3.4	0.003	0.39

In Minnesota, average rainfall in June and July is 102 and 99 mm while that in August and September is only 85 and 74 mm (weatherdb.com). Since rainfall during anthesis was the most significant term in determining yield, the manipulation of sowing time can be used to time echium growth with rainfall during anthesis, thereby increasing yield. Consequently, sowing echium in May, and assuming 45 days to first flower, synchronizes anthesis with higher summer rainfall and would be expected to achieve highest combined flower production and seed yield.

Echium yields for several experiments across North Dakota averaged 251 kg ha^−1^ and were as high as 425 kg ha^−1^ [10]. Target sowing dates for these experiments were late May, and growing season durations ranged from 77 to 99 d, whereas durations for the current experiments ranged from 89 to 112 d (table 1). In contrast, weedy echium growing in an ungrazed pasture in southern NSW produced over 30000 seed m^−2^ and had a 100-seed weight of 0.38 g [19]. These values convert to a seed yield of approximately 1100 kg ha^−1^. Plants in nearby grazed and mowed pastures produced about half of this amount (500 and 650 kg ha^−1^). These Australian seed production values included seeds retained on harvested plants as well as those in the litter and soil (2.5 cm depth). The average overall seed yield for the current experiments of 330 kg ha^−1^ is much lower than that for southern Australia, but higher than that for North Dakota, and it was above the minimum production goal of 250 kg ha^−1^ (see below).

Corn and soybean are the two main crops in the Upper Midwest and Northern Great Plains. Median gross returns (N = 2553 and 2209 farms) for these crops in 2012 were $2776 and $1628 ha^−1^, whereas net returns were $894 and $526 ha^−1^ according to the Farm Financial Database for Minnesota and the surrounding region (http://www.finbin.umn.edu). Net returns represented 32% of gross returns for both crops. To garner attention by growers, echium likely would have to match the net returns of corn or soybean. Assuming that the gross return to net return ratio is the same for echium as for corn and soybean, and that the contract price for echium seed is $5.30 kg^−1^ [9], then echium seed yields would need to be 310 kg ha^−1^ to match the net return of soybean and 527 kg ha^−1^ to match corn in 2012. Corn and soybean prices in 2012 were uncommonly high, thus an echium seed yield of 250 to 300 kg ha^−1^ probably represents a reasonable minimum production goal for profitability and grower acceptance.

## Conclusions

When sown in April or May in the Upper Midwest and Northern Great Plains, oilseed echium produces high seed yields whose value can rival those of standard commodity crops. With echium seed valued at $5.30 kg^−1^, early sown echium crops in 2011, 2012, and 2013 would have generated gross returns of $3710, $1733, and $2279 ha^−1^, respectively. In 2010, 2011 and 2012 the average gross returns for corn were $2020, $2260 and $2776 ha^−1^ (http://finbin.umn.edu), which overall makes echium a seemingly viable alternative crop from an economic perspective. In addition to its economic value, oilseed echium provides valuable ecosystem services (floral resources for pollinators) that cannot be matched by crops such as corn, soybean, and wheat. Early sown echium had high insect visitation and did not suffer the yield losses of late sown crops. From an agro-ecosystem perspective, early sown echium offers a high yielding crop to farmers and provides a valuable forage resource for pollinating insects.
